# Formation Mechanisms, Molecular Pathways, Mitigation Strategies, and Indoor Safety Risk Analysis of Cooking Oil Fumes

**DOI:** 10.3390/foods15111904

**Published:** 2026-05-28

**Authors:** Zhenkun Wang, Jingnan Chen, Wei Liu

**Affiliations:** 1 College of Food Science and Technology, Henan University of Technology, Lian Hua Street 100, Zhengzhou 450001, China; wangzhenkun106@163.com (Z.W.); chenjingnan813@126.com (J.C.); 2 National Engineering Research Center of Wheat and Corn Further Processing, Henan University of Technology, Lian Hua Street 100, Zhengzhou 450001, China

**Keywords:** cooking fumes, edible oils, human health risks, influencing factors, risk assessment

## Abstract

Cooking oil fumes (COFs) are major pollutants generated during thermal food processing, with emissions rising rapidly due to urbanization and the expanding catering industry, posing significant risks to indoor air quality and human health. This review systematically examines the formation mechanisms, physicochemical properties, and environmental and health impacts of COFs. Their formation involves primary processes such as thermal oxidation, cracking, Maillard reactions, and water vaporization, alongside secondary reactions where volatile organic compounds (VOCs) contribute to ozone (O_3_) and secondary organic aerosol (SOA) formation. COFs exhibit complex gas–liquid–solid coexistence and contain hazardous components including polycyclic aromatic hydrocarbons (PAHs), benzene compounds, aldehydes, and ultrafine particles (Dp ≤ 0.1 μm). Based on reported data, emission factors under typical cooking conditions range from 17.966 to 71.923 mg/(min·kg oil) for VOCs, 0.016 to 1.710 mg/(min·kg oil) for benzene compounds, and 0.458 to 1.820 mg/(min·kg oil) for formaldehyde. This highlights the variability in cooking fume emissions and associated health risks. Despite growing research attention, challenges remain in emission characterization and health risk assessment. By synthesizing current knowledge, this review provides a scientific basis for developing precise mitigation strategies and guiding future regulatory standards, with implications for improving food processing practices and indoor air quality management.

## 1. Introduction

The use of heated oils for cooking raw ingredients is a fundamental method of thermal food processing. In ancient China, early forms of pan-frying meats, often using animal fats, date back to the Zhou Dynasty (1046–256 BC), as documented in classical texts such as the Rites of Zhou (Zhouli), which distinguished between solid animal fats and liquid animal oils [1,2]. The use of heated oils allows for the rapid preparation (5–20 min) of various ingredients at high temperatures. This process yields food with enhanced color, aroma, flavor, and texture while simultaneously improving nutritional value and effectively eliminating harmful biological contaminants [3,4]. Currently, oil-based cooking is not only a primary method of household food preparation but also a key technique in industrial food production, such as in the manufacturing of fried instant noodles. COFs represent a significant pollutant generated during food processing in both domestic and commercial settings. Their complex chemical composition and potential toxicity constitute significant hazards to the atmospheric environment and human health. With the acceleration of urbanization and the rapid development of the tertiary industry, COFs from culinary sources have become a major unorganized contributor to urban air pollution, particularly in Chinese culinary methods, including high-temperature frying and stir-frying [5]. These methods significantly increase emissions of VOCs, particulate matter (PM), and PAHs. These pollutants not only directly harm the human respiratory and cardiovascular systems but also contribute to atmospheric photochemical reactions, leading to the formation of ozone (O_3_) and SOA [6]. SOA is formed through the transformation of atmospheric VOCs via processes such as multi-step oxidation, gas-particle partitioning, and particle-phase reactions. Specifically, VOCs undergo gas-phase oxidation under the action of oxidizing agents, generating a series of semi-volatile or low-volatility oxygenated organic compounds; these products subsequently enter the particle phase through gas-particle partitioning and undergo further condensation, polymerization, or liquid-phase reactions, ultimately forming SOA. Given that cooking fume emissions contain abundant VOCs, they are likely to undergo the aforementioned transformation processes in the atmosphere, leading to the formation of O_3_ and SOA [7]. This paper will discuss the composition of cooking fumes, the formation pathways of their hazardous components, and the secondary reaction mechanisms. The following discussion will first outline the chemical composition of cooking fumes, followed by an analysis of the formation pathways of hazardous components and the secondary reaction mechanisms of SOA. Based on this, the health risks of cooking fumes to humans will be examined, and the influencing factors and control strategies will be summarized.

The formation of COFs primarily stems from primary emission processes. Under high-temperature conditions, the unsaturated fatty acids in cooking oils participate in a cascade of reactions, including thermal oxidation, cracking, and β-scission. Concurrently, aldehydes and ketones, among other VOCs, are formed via Maillard reactions involving reducing sugars and amino compounds present in ingredients [8]. The physicochemical characteristics of COFs manifest as a mixed pollution profile featuring multi-phase (gas, liquid, and solid phases) and multi-scale (from PM_10_ down to nano-sized particles) properties. Chemically, COFs are primarily composed of aliphatic compounds (40–60%), though aromatic compounds (e.g., PAHs) and aldehydes have drawn significant attention due to their strong carcinogenicity. Although research on the environmental and health risks of COFs has made progress, several critical issues remain unresolved: (1) Systematic data on the emission patterns of Chinese-style cooking fumes and their influencing factors (such as regional dietary habits) are still lacking. Specifically, insufficient research has been conducted on the variations in cooking oil types (e.g., vegetable and animal oils) and ingredient combinations across different regional populations. The scarcity of long-term emission monitoring data in domestic cooking settings significantly hinders the accurate quantification of their pollution contribution. (2) Health risk assessments largely rely on external exposure levels, whereas the understanding of internal exposure doses and molecular mechanisms remains limited. (3) Existing control technologies exhibit low removal efficiency for ultrafine particles (PM_0.1_) and polar VOCs, which are often poorly suited for household applications. These limitations impede the development of precise and standardized COF pollution control systems.

This paper provides a systematic review of the formation mechanisms, physicochemical properties, environmental and health impacts, and control strategies of COFs, aiming to achieve the following objectives: (1) to elucidate key pathways of primary emissions and their environmental behaviors; (2) to analyze the relationship between chemical composition and toxic effects, providing a basis for health risk assessment; (3) to assess the health risks posed by COFs to humans; (4) summarize the influencing factors and regulation strategies for COFs generation; (5) to identify the inadequacies of current indoor safety standards when applied to kitchen environments by bridging fundamental research and standard-setting, and to propose directions for future revision. This review integrates insights from multiple disciplines to establish a scientific foundation for the precise control, standard formulation, and health intervention of COFs, with the ultimate goal of advancing efficient and sustainable pollution management in both household and commercial cooking fume pollution.

## 2. Physical and Chemical Features of Cooking Oil Fumes

**Figure 1:**
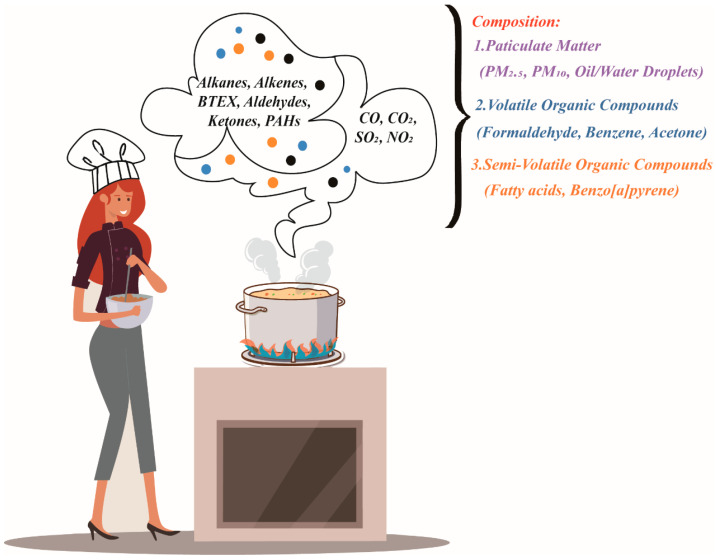
Physicochemical Composition of Cooking Oil Fume. Note: From a chemical perspective, the typical hazardous components include inorganic gaseous pollutants, benzene series compounds, carbonyls, and PAHs. From a physical perspective, COF comprises gaseous, liquid, and particulate phases, encompassing volatile and semi-volatile fractions.

As illustrated in Figure 1, COFs represent a complex mixture generated during cooking processes, and their physicochemical composition is influenced by multiple factors. The emissions consist of both gas-phase compounds and particulate matter, with the latter potentially containing a combination of liquid and solid components. These components encompass a wide range of chemical species. In addition to their intrinsic chemical toxicity, these compounds may undergo atmospheric reactions to form secondary pollutants, posing significant implications for indoor air quality and human health.

### 2.1. Physical Morphology and Size Distribution of Cooking Oil Fume

From a physical perspective, the particulate matter emitted during cooking consists primarily of condensed-phase particles accompanied by VOCs in the gas phase. COFs represent a mixed pollution system characterized by the coexistence of gas-phase species and particulate matter, with the latter containing both solid and liquid components [9,10]. The liquid fraction exhibits high viscosity. The overall particle size distribution is relatively fine, with aerodynamic diameters (Dp) typically below 10 μm. According to particle size, these emissions can be classified into inhalable particles (PM_10_, Dp ≤ 10 μm), fine particles (PM_2.5_, Dp ≤ 2.5 μm), submicron particles (PM_1.0_, Dp ≤ 1.0 μm), and ultrafine particles (UFPs, Dp ≤ 0.1 μm) [5,11,12,13]. This multi-phase and multi-scale physical nature endows COFs with significant specificity and complexity in terms of atmospheric transport, transformation, and health impact mechanisms.

### 2.2. Chemical Composition and Classification of Cooking Oil Fumes

COFs are a complex mixture comprising gas-phase compounds (e.g., VOCs and volatile fractions of SVOCs) and particulate matter (including semi-volatile species partitioned into the condensed phase). In recent years, extensive research has systematically analyzed their chemical composition. Current findings reveal that COFs exhibit remarkable chemical diversity, primarily including aliphatic compounds (e.g., alkanes, alkenes, and fatty acids), oxygenated organic compounds (e.g., aldehydes, ketones, alcohols, esters), aromatic compounds (e.g., benzene-series compounds, PAHs), and heterocyclic compounds [14,15,16]. Regarding compositional distribution, aliphatic compounds (alkanes, alkenes, and fatty acids) account for the highest proportion (approximately 40–60%), which is closely related to the main components of cooking oils. Following these are oxygenated organic compounds (e.g., aldehydes, ketones, esters), which constitute approximately 20–30% of COFs. Although aromatic and heterocyclic compounds constitute a relatively smaller share (10–15%), they have garnered considerable attention owing to their pronounced toxicity and carcinogenicity [17,18,19,20,21].

Regarding the composition of particulate matter, the chemical profiles of PM_2.5_ from cooking emissions have been extensively analyzed in various studies. Research by Li et al. demonstrated that organic carbon constitutes the dominant constituent of cooking-derived PM_2.5_, representing 41.67–57.91% of its mass, followed by elemental carbon, water-soluble ions, and heavy metals (e.g., copper, zinc, lead, etc.) [22]. It is noteworthy that due to the fine particle size and large specific surface area of particles generated during cooking [23], various harmful components in cooking oil fumes are readily adsorbed onto the PM_2.5_ surface. Xu et al. reported that the mass concentration of organic compounds (including alkanols, alkanes, carboxylic acids, and PAHs) in cooking-source PM_2.5_ ranged from 1112 to 32,016 ng·m^−3^, representing on average 11% of the PM_2.5_ mass [24].

## 3. Formation Pathways and Mechanisms of Cooking Oil Fumes

**Figure 2:**
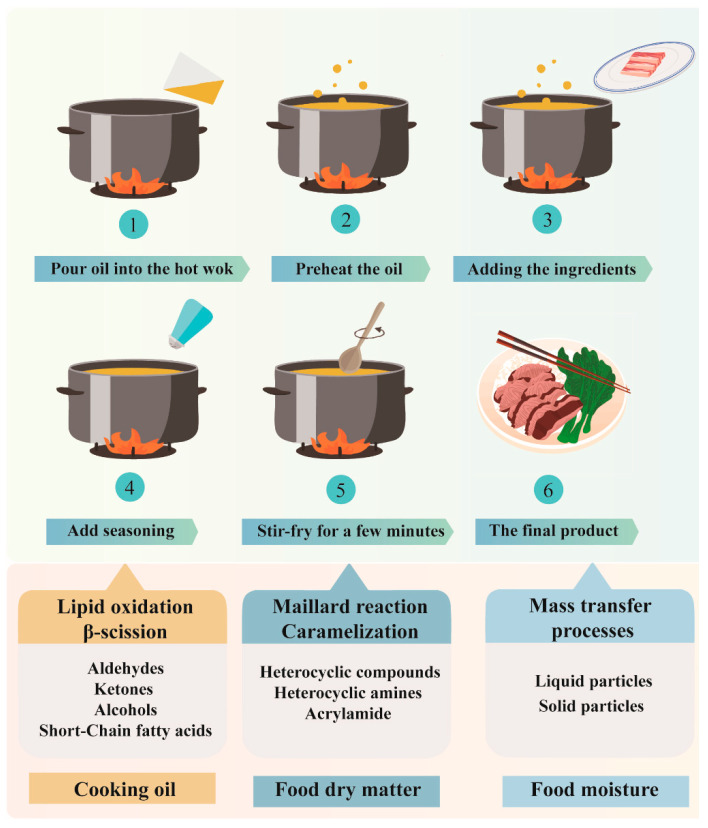
Chemical reactions and their concomitant hazardous substances in Chinese cuisine preparation.

In recent years, systematic studies on VOC pollution sources in China have shown the following trends. Conventional pollution sources, such as vehicle exhaust, industrial emissions, and coal combustion, have been increasingly controlled. At the same time, the service industry has expanded rapidly. As a result, the contribution of unorganized emission sources to atmospheric VOCs has significantly increased. Among these, cooking emissions, as a major unorganized source in urban areas, have emerged as one of the critical determinants of urban air quality [25,26]. The generation mechanism of COFs involves multi-stage physicochemical transformation processes, as illustrated in Figure 2. Initially, cooking oil is composed primarily (over 95%) of triglyceride fatty acids. During heating, it undergoes complex thermal chemical reactions. These reactions include thermal oxidation in the 100–270 °C range. In this process, unsaturated fatty acids undergo autoxidation and generate hydroperoxides. These hydroperoxides further convert into VOCs, such as aldehydes and ketones. This produces the characteristic “blue smoke” [3]. β-scission reactions occur at high temperatures (>270 °C). They lead to the breakdown of the glycerol backbone and generate irritant compounds such as acrolein. In addition, trace components in the cooking oil undergo volatilization and oxidation. Secondly, after ingredients are added, rapid water vaporization induces mass transfer processes. The Maillard reaction, a thermal reaction between reducing sugars and amino acids, further promotes the formation of oxygenated organic compounds and nitrogen-containing heterocyclic compounds. These gaseous products subsequently condense. This forms an aerosol system dominated by particulate matter. Eventually, these become visible oil fumes [27,28,29]. Moreover, reactive VOCs released during cooking serve as important precursors in atmospheric photochemical reactions, resulting in ozone and SOA production through complex gas-phase oxidation pathways [30]. Therefore, in-depth research on the formation mechanisms of cooking oil fumes is of great significance for pollution control and mitigation.

Note: This figure illustrates how cooking operations (heating cooking oil, adding ingredients and seasonings, stirring/tossing) trigger chemical reactions involving cooking oil, food components, and food moisture during thermal processing, thereby generating risk components. The specific mechanisms are as follows: cooking oil undergoes thermal oxidation and β-cleavage at high temperatures; food components (e.g., proteins, carbohydrates) participate in Maillard reactions and caramelization; food moisture promotes hydrolysis reactions and the steam-mediated release of volatile organic compounds.

### 3.1. Primary Emission Pathways

The primary emission pathways of COFs encompass the thermal oxidative cracking of edible oils, the Maillard reaction occurring in food ingredients, and the mass transfer induced by moisture vaporization. These mechanisms directly contribute to the formation and release of VOCs, SVOCs, and PM.

#### 3.1.1. Thermal Oxidation of Edible Oils

Thermal oxidation is a complex chemical reaction that occurs in cooking oil under high temperatures (typically > 120 °C) under oxygenated conditions [31]. This process encompasses free radical chain reactions [32], cleavage, polymerization, and hydrolysis [33]. Unlike autoxidation at room temperature, thermal oxidation is more intense and rapidly generates harmful products [34].

**Figure 3:**
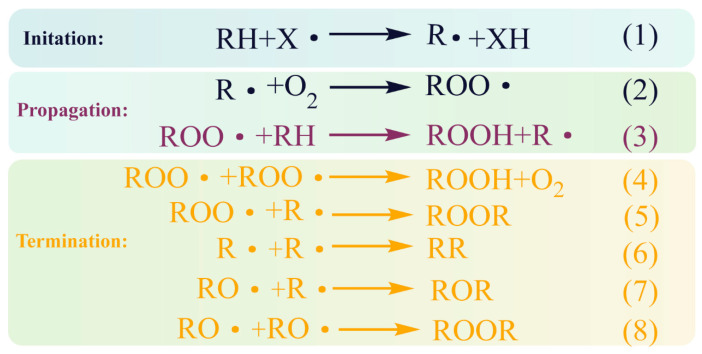
Thermal oxidation mechanism of edible oils. Note: This reaction consists of three stages: chain initiation, chain propagation, and chain termination. In the figure, RH represents the unsaturated substrate participating in the reaction, and H represents the most active hydrogen atom on the methylene group adjacent to the double bond.

As shown in Figure 3, unsaturated fatty acid molecules contain carbon-carbon double bonds. Due to these bonds, the hydrogen atom at the allylic position (α-position of the double bond) has a low bond dissociation energy. This hydrogen is therefore highly reactive. Under thermally initiated conditions, initiators in the system (such as radical initiators or metal ions) readily abstract these reactive hydrogens. This process generates alkyl radicals (R•). These alkyl radicals (R•) rapidly undergo auto-oxidation to form peroxyl radicals (ROO•). These ROO• then abstract hydrogen atoms from neighboring unsaturated fatty acid molecules. This generates hydroperoxides (ROOH) and simultaneously produces new alkyl radicals (R•). This chain reaction is thus sustained. The resulting hydroperoxides (ROOH) have poor thermal stability. They decompose into low-molecular-weight volatile compounds, such as aldehydes and ketones [35,36]. Meanwhile, the oxidative polymerization of fats and oils occurs primarily through radical termination reactions. In these reactions, radicals combine to form dimers and polymers. This leads to increased viscosity and darkening of color. These polymers also affect the edible quality of the oil [37].

#### 3.1.2. Hydrolysis of Triglycerides

**Figure 4:**
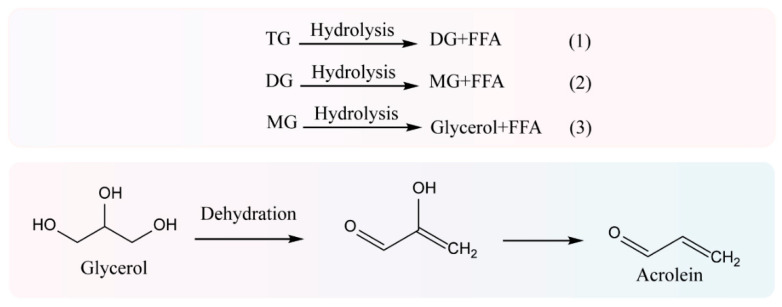
Hydrolysis process and products of edible oils. Note: The main component of cooking oil is triglycerides (TG). In the presence of moisture, TG is progressively hydrolyzed into diglycerides (DG), monoglycerides (MG), and free fatty acids (FFA). The generation of FFA further exacerbates the thermal oxidation of cooking oil. The end product, glycerol, can be converted into the carcinogen acrolein via dehydration.

As shown in Figure 4, TG can undergo hydrolysis with moisture from food ingredients under high-temperature conditions. This reaction produces DG, MG, FFA, and glycerol. The resulting FFA can accelerate fat and oil deterioration. They can also promote oxidative reactions. Glycerol is the end product of hydrolysis. It can be further converted into acrolein, a known carcinogen, through a dehydration reaction. The oxidation of fatty acids and the hydrolysis of glycerol lead to a decline in oil quality. They also promote the formation of VOCs and polar compounds, such as aldehydes, ketones, and acids [38,39,40]. Under high-temperature conditions, these products can participate in aerosol formation or polymerization reactions. This process promotes the generation of COFs and other complex organic particulate matter.

#### 3.1.3. β-Scission of Unsaturated Fatty Acids

**Figure 5:**
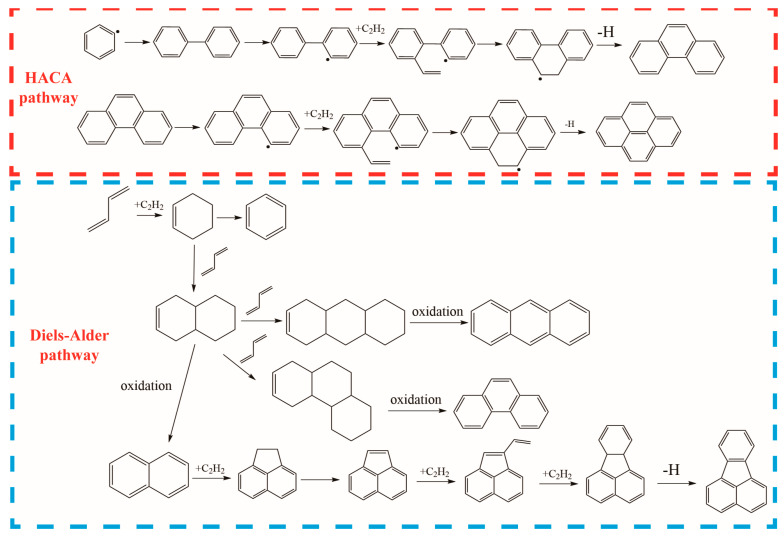
The generation mechanism diagrams of the HACA pathway and the Diels-Alder pathway for polycyclic aromatic hydrocarbons PAHs.

**Figure 6:**
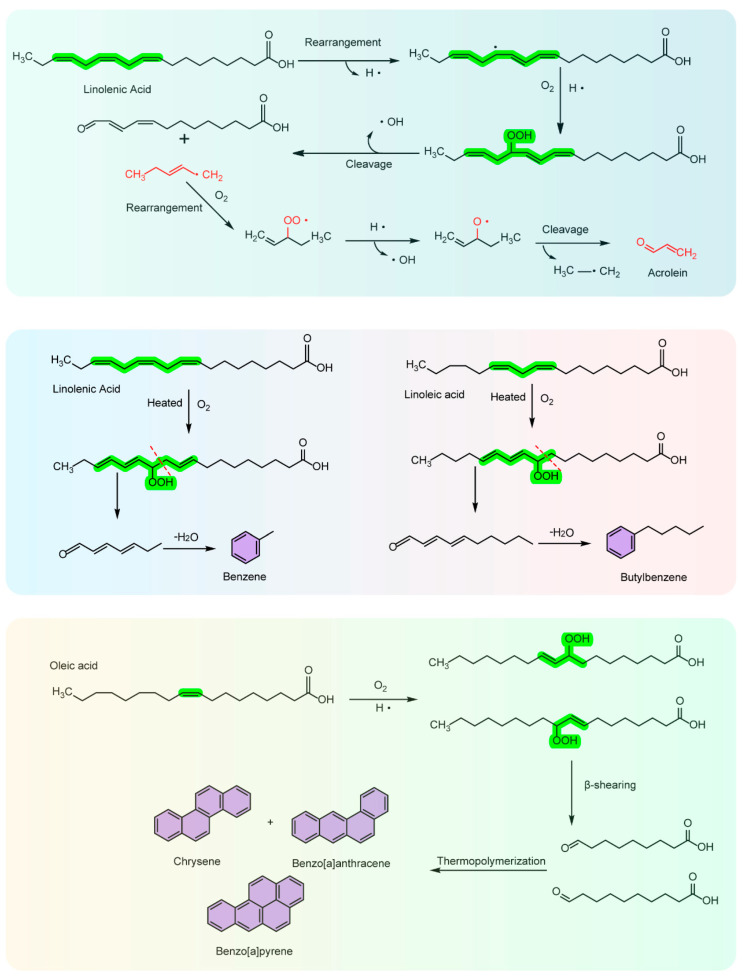
Formation mechanisms of typical hazardous components (acrolein, benzene, and PAHs) in cooking oil fume. Note: The figure systematically outlines the key reaction mechanisms by which representative polyunsaturated fatty acids in edible oils form low-molecular-weight aldehydes (acrolein), benzene derivatives (toluene, styrene), and PAHs under high-temperature conditions through reaction pathways such as thermal oxidation, β-scission, and molecular rearrangement.

Figure 5 summarizes the generation mechanism of PAHs under high-temperature conditions. On this basis, Figure 6 illustrates the β-scission reaction of unsaturated fatty acids in cooking oils under high-temperature or oxidative conditions, which is a free radical-mediated chain cleavage process. This process results in the decomposition of fatty acid chains, yielding low-molecular-weight volatile compounds, which not only affect the flavor and safety of the oil but also constitute important components of COFs [41,42]. When exposed to heat or catalytic metal ions, unsaturated fatty acids (RH) in cooking oils are oxidized. This process yields alkyl radicals (R·). The oxygen atom in alkoxy radicals (RO·) is highly electronegative and unstable. It strongly favors electron acquisition to achieve a stable structure. This electronic state propagates through the carbon chain. It significantly weakens the C-C bond between the α-carbon (directly bonded to oxygen) and the β-carbon (adjacent carbon). This makes the bond exceptionally fragile and prone to cleavage [43,44,45,46,47].

#### 3.1.4. Contributions of Maillard Reaction to Cooking Oil Fume Generation

During cooking, primary emissions from ingredients mainly originate from various chemical reactions under high-temperature conditions. The Maillard reaction serves as a representative example, entailing a cascade of reactions between carbonyl compounds (primarily reducing sugars) and amino compounds (including amines, amino acids, peptides, and proteins) during heating [48]. Three stages could be identified in this reaction. The first stage condensation of the carbonyl group (=CO) of reducing sugars with the amino group (-NH_2_) leads to the formation of an unstable Schiff base, followed by intramolecular rearrangement to yield stable Amadori rearrangement products (for aldoses) or Heyns rearrangement products (for ketoses). At the intermediate stage, the rearrangement products are further converted via pathways such as 1,2-enolization, 2,3-enolization, and Strecker degradation. These reactions generate key intermediates including furans, reductones, dehydroreductones, and Strecker aldehydes (e.g., phenylacetaldehyde, methylpropanal) [49]. In the concluding phase, these intermediates proceed to polymerize or condense, affording melanoidins and a range of heterocyclic compounds [49,50,51]. Significantly, aside from imparting characteristic flavors, the Maillard reaction serves as a key driver of cooking organic aerosol formation by generating carbonyl compounds.

The Maillard reaction bears a profound and complex relationship with cooking fumes. While it is essential for flavor development, it also constitutes a significant source of indoor air pollutants, specifically through its generation of organic particulate matter [52,53]. Driven by the thermodynamics of high-temperature cooking, the Maillard reaction directly generates large quantities of volatile carbonyl compounds, heterocyclic amines, and semi-volatile organic compounds. These products, formed via the condensation, degradation, and polymerization of amino acids and reducing sugars, subsequently act as condensation nuclei for aerosol particles. Moreover, it deeply intersects with concurrent lipid thermal oxidation and hydrolysis reactions, resulting in mutual catalysis and forming a complex reaction network [54,55,56]. This intricate system collectively dictates the aerosol composition, physicochemical properties, and ultimately the health risk profile of cooking fumes. For example, heterocyclic amines—specific products of the Maillard reaction—could adsorb onto particles. Once inhaled, these particle-bound compounds present potential respiratory and carcinogenic risks [49]. Consequently, the Maillard reaction serves as a key driver in the chemical formation of oil fume aerosols, holding essential scientific significance for accurate exposure risk assessment and the development of source reduction and control strategies.

#### 3.1.5. Influence of Food Moisture Vaporization on Cooking Oil Fume Formation

During cooking, the evaporation of water from ingredients is not only a physical change but a significant mass transfer process. In this process, water vapor acts as a carrier that entrains volatile and semi-volatile hazardous substances (such as PAHs and heterocyclic amines) from oils into the gas phase, thereby considerably influencing indoor air quality and human exposure levels. This mechanism is analogous to distillation. Its mass transfer efficiency is regulated by multiple factors including temperature, water content, oil type, and ingredient surface area [5]. Thermal cooking methods (e.g., frying and deep-frying) particularly exacerbate the release of VOCs and particulate matter (PM_2.5_). Water vapor can facilitate the co-volatilization of polar compounds while simultaneously influencing the gas–particle partitioning of pollutants via condensation mechanisms [56]. A deep understanding of the mass transfer mechanisms and pollutant migration during water evaporation is crucial for accurately assessing exposure risks from cooking emissions and developing efficient source control strategies [57].

### 3.2. Secondary Transformation Processes of Cooking Oil Fumes

VOCs emitted during cooking undergo significant secondary transformations in the atmosphere. These transformations primarily generate O_3_ and SOA through photochemical reactions [58,59]. GC-MS analysis indicates that these VOCs primarily include aromatic hydrocarbons, aldehydes, ketones, halogenated alkanes, alkenes (e.g., α-pinene and terpenes), and alkanes [60,61]. Among these, monoterpenes (e.g., α-pinene and β-pinene) are important precursors for O_3_ and SOA. Their oxidation processes generate various oxygenated organic compounds. In addition, compounds such as isoprene also contribute significantly to SOA formation through photooxidation [62].

The SOA formation mechanism for cooking fume VOCs is as follows. First, these VOCs undergo gas-phase oxidation reactions with atmospheric oxidants such as OH, O_3_, or NO_3_. These reactions produce semi-volatile or low-volatility intermediates, including oxygenated organic compounds and peroxy radicals. Taking monoterpenes as an example, their oxidation can form aldehydes, ketones, and carboxylic acids. These products have reduced volatility. They readily enter the existing organic particulate phase through gas-particle partitioning [7]. Second, some of these products undergo heterogeneous oxidation or liquid-phase reactions on cloud or fog droplet surfaces. This further generates low-volatility substances. Organic compounds that enter the particulate phase can also undergo oligomerization reactions. These include aldehyde-alcohol condensation and esterification. Such reactions form high-molecular-weight SOA components [63]. This process is significantly regulated by NOx concentration, relative humidity, and particle acidity. For example, high NOx conditions often promote nitro compound formation. This alters the chemical composition and yield of SOA [64]. Carbonyl compounds (aldehydes and ketones) participate in atmospheric chemical processes. They act both as reaction products and as precursors for further reactions. Aromatic hydrocarbons influence atmospheric oxidation capacity through specific reaction pathways [65]. These secondary transformation processes significantly impact regional air quality.

### 3.3. Multistage Reaction Pathways in the Formation of Cooking Fumes

**Figure 7:**
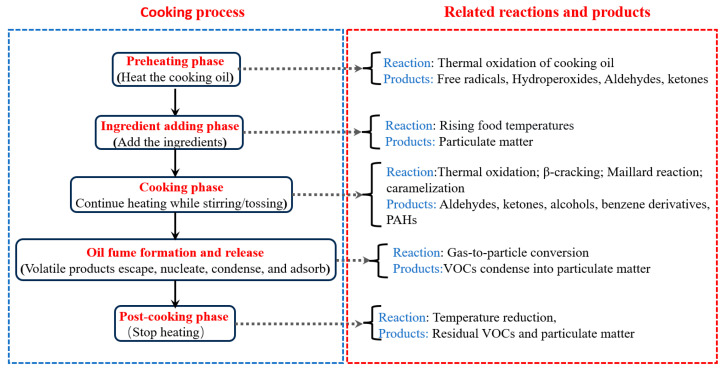
A unified conceptual framework for the formation of cooking fumes. Note: This figure divides the cooking process into five key stages. It illustrates the temporal relationships and products of thermal oxidation, hydrolysis, β-cracking, and the Maillard reaction, thereby establishing a complete pathway from cooking operations to the generation of cooking fumes.

As shown in Figure 7, based on the sequence of cooking operations, the formation process of COFs can be divided into five stages. First is the preheating stage. Before ingredients are added, the cooking oil is heated alone. The oil temperature gradually rises from room temperature to approximately 120–180 °C [31]. This is the chain-initiation stage of thermal oxidation. The unsaturated fatty acids in the oil produce small amounts of free radicals and primary oxidation products, such as hydroperoxides. This stage provides active precursors for subsequent reactions [32]. However, the amount of smoke released is relatively low. Second is the ingredient addition stage. Moisture-containing ingredients and seasonings are added to the hot oil. The local temperature drops sharply and then rises rapidly. Moisture on the ingredient surface vaporizes quickly. Moisture also penetrates the oil phase. This induces triglyceride hydrolysis, which produces FFAs [38]. Proteins and reducing sugars in the ingredients come into contact with the oil. They provide substrates for the Maillard reaction. This stage creates the initial conditions for the Maillard reaction. Third is the cooking stage. Under continuous heating and stirring, the oil temperature is typically maintained between 150 °C and 250 °C. This is the most chemically complex period. Thermal oxidation enters the chain transfer and branching stages. These stages produce large amounts of free radicals and peroxides. The FFAs generated by hydrolysis further accelerate thermal oxidation reactions. Thermal oxidation intermediates undergo β-scission. This forms small-molecule VOCs, including aldehydes and benzene derivatives. Simultaneously, the Maillard and caramelization reactions of food components continue. They generate hazardous compounds, such as PAHs [25,26,27]. These reaction pathways intertwine. They form a complex reaction network. Fourth is the formation and release stage of cooking fumes. Volatile reaction products accumulate. Small-molecule VOCs then escape from the oil phase into the gas phase. Some VOCs condense to form particulate matter; others adsorb onto particle surfaces. Gaseous and particulate components together form cooking fumes [29]. These fumes are released into the surrounding environment. This stage is a critical component of cooking fume exposure assessment. Fifth is the post-cooking stage. After heating stops, residual heat keeps the oil temperature high for some time. Remaining free radicals and reaction intermediates undergo slow oxidation and cracking reactions. Some volatile products are also released.

## 4. Environmental Impact and Assessment Models of Cooking Oil Fumes

### 4.1. Atmospheric Environmental Impact of Cooking Oil Fumes

An aerosol refers to a relatively stable suspension resulting from the uniform dispersion of liquid or solid particles within a gaseous medium. On the basis of their formation mechanisms, atmospheric aerosols may be subdivided into two categories: primary aerosols and SOA. Primary aerosols originate directly from emission sources as primary particulate matter [66]. Secondary aerosols are formed when atmospheric VOCs undergo gas-phase photo-oxidation or oxidation by oxidants. These reactions generate semi-volatile organic compounds. These compounds then convert into solid or liquid particles through gas-particle partitioning. These secondary aerosols are a major component of atmospheric particulate matter [67].

As a significant source of indoor pollution, COFs have dual impacts. Their volatile components cause noticeable irritant effects indoors, while also influencing the regional atmospheric environment as a source of secondary pollutants. Studies have shown that cooking organic aerosols contain substantial amounts of PM_2.5_ [68,69]. Characterized by small particle size and large specific surface area, these particles are prone to prolonged atmospheric suspension, long-range transport, and strong adsorption of toxic substances, particularly pathogenic microorganisms and heavy metals. More importantly, COFs serve as important precursors for haze formation [70]. Furthermore, VOCs emitted from cooking fumes are involved in photochemical pathways with atmospheric nitrogen oxides (NOx), thus contributing to the formation of ozone, SOA, and haze. This process significantly influences regional atmospheric air quality [71].

#### 4.1.1. Emission Characteristics of Key Pollutants from Cooking Oil Fumes

The characteristic pollutants in COFs are primarily particulate matter, carbonyl compounds (aldehydes and ketones), aromatic hydrocarbons, and PAHs, all four types of which exhibit distinct emission profiles. According to the study by Kang et al., the emissions of PM_2.5_ generated from cooking range from 2.14 to 22.84 mg/min, and the emissions of PM_10_ range from 2.35 to 25.57 mg/min. The emissions of PM_10_ are 9.8% to 12.0% higher than those of PM_2.5_ [72]. Additionally, in a study of catering establishments in Guangzhou, the total concentration of carbonyl compounds (as aldehydes) was monitored in the range of 92.9 to 581 μg/m^3^ [73]. Formaldehyde accounted for the highest proportion (30–45%), which might be attributed to its relatively low thermal stability. Furthermore, low-molecular-weight carbonyls (C_1_–C_3_) constituted a significant majority (63–96.08%) of the total carbonyl compounds. This distribution pattern is likely associated with differences in the volatility of various carbonyl species [74].

The emission profiles of BTEX (benzene, toluene, ethylbenzene, and xylenes) are strongly governed by both the type of cooking oil and the heating temperature. In controlled experiments conducted by Zhang et al., marked variations in BTEX emissions were observed among different cooking oils (p<0.05), with emission rates following the descending order of: toluene (0.051–0.171 mg/min) > xylene (0.007–0.034 mg/min) > benzene (0.0002–0.013 mg/min). Notably, when the cooking temperature exceeded 200 °C, the emission of BTEX increased significantly, with increments ranging from 35% to 72%. This phenomenon is possibly related to the intensified thermal oxidative decomposition of cooking oil at high temperatures [73].

PAHs are important components in catering-derived particulate matter with strong carcinogenicity. The study by Xiao et al. demonstrated that the total concentration of 16 PAHs in catering-source PM_2.5_ ranged from 0.04 to 0.24 μg/m^3^, with benzo[g,h,i]perylene (18.0%), pyrene (17.8%), and phenanthrene (12.8%) identified as the dominant monomers. In terms of ring size distribution, 4-ring PAHs exhibited the highest proportion (29.2–52.4%), followed by 5–6 ring PAHs (23.1–38.5%) and 2–3 ring PAHs (18.3–32.4%). This characteristic may be attributed to the mechanisms of high-temperature cracking and thermal polymerization of organic compounds during cooking processes [75].

#### 4.1.2. Comparison of TVOC Emission Intensities from Cooking Fumes

A comparison of TVOC concentration ranges among Chinese cooking, industrial emissions, and urban traffic reveals the following trends. Chinese cooking (233.97–483.16 μg/m^3^) has significantly higher concentrations than urban traffic (72.85–131.48 μg/m^3^). However, its overall emission intensity is lower than that of industrial emissions (326.67–2391.33 μg/m^3^). In particular, the upper limit of industrial emissions exceeds that of cooking emissions by nearly five times. This indicates that urban industrial emissions may still be the primary source of VOCs [76,77,78].

However, the concentration range for Chinese-style cooking is relatively narrow. This may result in stable, moderate-intensity pollution during specific time periods (e.g., peak meal times) and in specific spaces (e.g., commercial kitchens) [76]. In contrast, urban traffic emissions have the lowest average values. However, they may exhibit instantaneous peaks in road environments due to traffic density and low-speed driving [78]. These differences show that the three source categories have distinct characteristics. These include emission intensity, temporal persistence, and spatial exposure patterns. Therefore, each category should be considered separately in future VOCs control strategies and relevant standards. This will enhance the effectiveness and specificity of pollution control.

#### 4.1.3. Assessment Models for Ozone and Secondary Organic Aerosol Formation

(1) Ozone Formation Potential

Ozone formation potential (OFP) is a key metric for evaluating the capacity of VOCs to generate ozone in photochemical reactions [26,79,80,81,82]. It is calculated based on the Maximum Incremental Reactivity (MIR) of each VOC species and its ambient concentration, using the following formula:

$$\begin{array}{l} OFP_{i}=VOC_{i}*MIR_{i}\\ OFP_{\mathrm{Tot}.}=\sum_{i=1}^{n}OFP_{i} \end{array}$$

where OFP represents the ozone formation capacity of a VOC species (unit: µg O_3_/m^3^); VOC denotes the concentration of the species (unit: µg/m^3^); and MIR represents the Maximum Incremental Reactivity of the VOC species (unit: g O_3_/g VOC, which is dimensionless).

(2) Secondary Organic Aerosol Formation Potential

The SOA formation potential refers to the ability of VOCs to transform into SOA through atmospheric photochemical reactions [61]. Current commonly used assessment methods in the literature primarily include empirical models and kinetic models. The details are shown below:

(1) Empirical Models Based on Aerosol Formation Coefficient

Empirical models estimate the SOA formation potential using the aerosol formation coefficient (FAC). The basic formula is as follows [80,81,83]:

$$\begin{array}{l} SOA_{p}=VOC_{S0}*FAC\\ SOA_{p,tot}=\sum_{i=1}^{n}SOA_{p,i} \end{array}$$

where *SOA_p_* denotes the potential secondary organic aerosol production mass; *VOC_s_*_0_ represents the initial volatile organic compound concentration; *FAC* refers to the aerosol formation coefficient.

(2) Kinetic Models Based on VOC–OH Reaction Kinetics

Kinetic models calculate the SOA formation mass based on the reaction kinetics between VOCs and hydroxyl radicals (·OH), considering each component individually [84,85]:

$$SOA=\sum [HC_{i}]*(1-e^{-K_{OH,i}*[OH]*\Delta t})*Y_{i}$$

where [*HC_i_*] denotes the initial concentration of the volatile organic compound, expressed in units of µg/m^3^ or mol/m^3^; *k_OH_* represents the rate constant for the reaction of the species with hydroxyl radicals (·OH); [*·OH*] refers to the concentration of hydroxyl radicals; Δ*t* represents the reaction time, in units of seconds (s); *Y_i_* stands for the SOA yield of component i; the term (1 − *e*^−*kOH*,*i*×^[*OH*]^×Δ*t*^) describes an exponential decay function, quantifying the fraction of VOC oxidized by ·OH over the time interval Δt.

## 5. Health Risks Associated with Cooking Oil Fume Exposure

**Figure 8:**
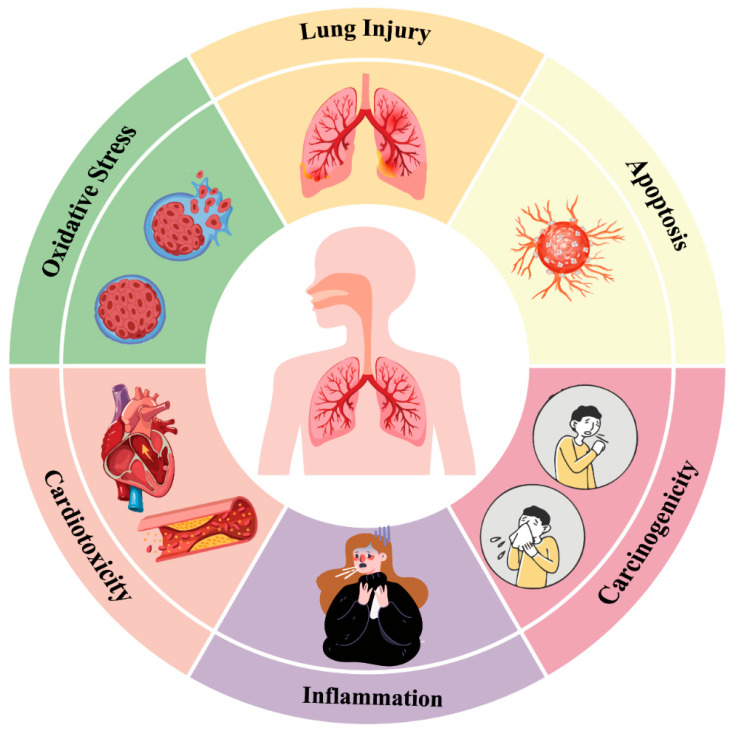
Major health risks associated with COF exposure.

Studies have indicated that organic aerosols generated from COFs contain multiple hazardous components that could cause multi-system damage to human health. As illustrated in Figure 8, major health risks include: respiratory system impairment [86], immunosuppression [87], reproductive toxicity [88], induction of DNA damage [89,90,91], promotion of reactive oxygen species (ROS) generation [90], triggering of apoptosis [92], cardiovascular diseases [93], and induction of inflammation [92].

### 5.1. Major Toxic Components in Cooking Oil Fumes

COFs contain multiple chemical constituents with significant toxicological implications. As summarized in Table 1, these include aromatic hydrocarbons, aldehydes, ketones, PAHs, and fine particulate matter.

### 5.2. Cooking Oil Fume Exposure Routes and Particulate Deposition Regions

The exposure pathways for COFs depend on their physical state and particle size distribution and are outlined below. Volatile components are mainly inhaled through the respiratory tract, while ultrafine particles can penetrate into the lungs and are then transported within the body [96].

Particulate matter exposure occurs through inhalation and dermal contact [97]. Inhalation exposure exhibits significant particle-size dependence: aerodynamic diameter (AD) > 50 μm, unable to be inhaled; 10–50 μm, primarily deposited in the nasal cavity; ≤10 μm, capable of entering the respiratory tract; ≤2.5 μm (PM_2.5_), could penetrate deeply into the alveolar region [98]. In addition, larger particles (>10 μm) may settle and cause exposure through dermal contact. These particles could be absorbed trans dermally or cause local irritation upon contact [99].

### 5.3. Penetration, Systemic Circulation, and Neurotoxicological Mechanisms of Ultrafine Particles

For PM_0.1_, the toxicological mechanisms are more complex than those of larger particles. They primarily involve three aspects: penetration capacity, systemic circulation, and neurological effects. First, due to its extremely small size, PM_0.1_ can enter the central nervous system via two pathways. The first pathway is through the alveolar epithelium or gastrointestinal tract into the bloodstream. After entering systemic circulation, a portion of these particles crosses the blood–brain barrier. The second pathway involves particles deposited on the olfactory mucosa in the nasal cavity. These particles are taken up by olfactory neuron terminals and transported retrogradely via axons directly into the olfactory bulb, bypassing the blood–brain barrier [96,100]. Oberdörster et al. conducted a rat inhalation study using isotope-labeled ^13^C ultrafine particles (36 nm). They demonstrated that particle concentrations in the olfactory bulb continued to rise within 7 days of exposure (from 0.35 μg/g to 0.43 μg/g). In contrast, only transient elevations were observed in the cerebral cortex and cerebellum. These findings suggest that the olfactory nerve pathway is the primary route for particle entry into the brain, while the bloodstream serves as a secondary route [96]. Second, PM_0.1_ that enters the bloodstream can accumulate in peripheral organs such as the liver and heart. This induces systemic inflammatory responses and may increase the risk of thrombosis [96,101]. Finally, PM_0.1_ transported directly to the olfactory bulb, affects neuronal mitochondria. This induces oxidative stress and local neuroinflammation. Long-term exposure may lead to tau protein hyperphosphorylation and Alzheimer’s disease [102]. Furthermore, PM_0.1_ in the systemic circulation can indirectly activate brain microglia. It does so by inducing the release of peripheral inflammatory cytokines (e.g., IL-6 and TNF-α). This establishes an indirect pathway of neurological damage [103]. In summary, the neurotoxicological mechanisms of PM_0.1_ involve dual pathways: direct neural transport and indirect systemic inflammatory effects. Both pathways may synergistically exacerbate damage to the central nervous system.

### 5.4. Health Risks of Cooking Oil Fume to Humans and the Underlying Mechanisms

**Figure 9:**
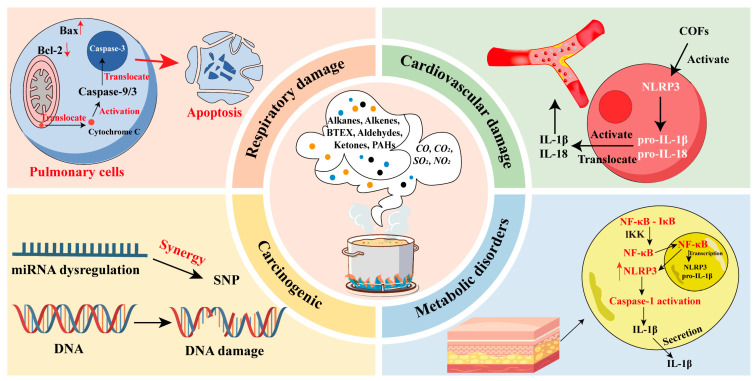
The molecular mechanisms underlying COF exposure-induced multi-system health risks. Note: This figure summarizes the associations between COF exposure and respiratory diseases, cardiovascular diseases, metabolic disorders, and cancer. Different colors represent the key nodes of each pathway and their corresponding outcomes.

COFs are a significant source of indoor air pollution due to their complex chemical compositions and associated health hazards. Prolonged exposure substantially increases health risks for affected individuals. As illustrated in Figure 9, understanding the underlying mechanisms of these adverse effects is critical for developing evidence-based intervention strategies and safeguarding public health.

(1) Mechanisms of Lung Injury and Apoptosis

Previous studies have confirmed that COFs are closely associated with lung damage. COFs are a major source of indoor air pollution. Epidemiological evidence suggests that COF exposure is a key risk factor for lung cancer [104]. Animal studies have shown that short-term COF exposure can lead to increased airway secretions, reduced lung tissue elasticity, and airway constriction. These changes thereby impair lung function [105]. At the molecular level, COF exposure disrupts the oxidative/antioxidant balance. This is evidenced by elevated levels of malondialdehyde (MDA) and reactive oxygen species (ROS), as well as decreased glutathione (GSH) activity. Oxidative stress activates the nuclear factor κB (NF-κB) signaling pathway. It also induces DNA damage. In addition, it promotes the release of inflammatory factors, such as tumor necrosis factor-α (TNF-α) and interleukin-1β (IL-1β). These factors trigger pulmonary inflammation. Furthermore, oxidative stress induces mitochondrial dysfunction. This dysfunction ultimately leads to alveolar epithelial cell apoptosis through the upregulation of caspase-3 expression [106].

Further studies have revealed that COFs can induce apoptosis in fetal alveolar type II epithelial cells (AEC II) through multiple pathways. Che et al. noted that COFs activate both the mitochondrial pathway and the death receptor pathway simultaneously [107]. In the mitochondrial pathway, COFs upregulate the pro-apoptotic protein Bax and inhibit the anti-apoptotic protein Bcl-2. They also alter mitochondrial permeability and promote cytochrome translocation to the cytoplasm and nucleus. Subsequently, they activate the caspase-9 and caspase-3 cascade. Concurrently, COFs increase caspase-8 levels by regulating the death receptor Fat. This leads to cell cycle arrest at the G0-G1 phase. These findings indicate that COFs induce apoptosis in AEC II by synergistically activating multiple apoptotic signaling pathways [97].

(2) Carcinogenicity

Both epidemiological and molecular biological studies have confirmed that COF exposure is closely associated with an increased risk of lung cancer among Chinese women. A case–control study by Liu et al. demonstrated that long-term COF exposure significantly increases lung cancer incidence in this population [108]. Wang et al. conducted a systematic review of the carcinogenic mechanisms of COFs [109]. These mechanisms include genetic damage induction, oxidative stress triggering, DNA repair inhibition, miRNA regulation disruption, and protein function impairment. Notably, Yin et al. found that single-nucleotide polymorphisms (SNPs) in five key miRNAs exhibit a significant synergistic carcinogenic effect with COF exposure [110]. The interaction between these genetic variants and environmental pollutants significantly increases lung cancer risk.

(3) Cardiovascular Damage and Dyslipidemia

PM_2.5_ in COFs can induce cardiovascular damage through specific molecular pathways [111]. Experiments by Ding et al. demonstrated that exposure to PM_2.5_ from COFs significantly elevates intracellular and mitochondrial ROS levels. This disrupts cellular redox balance. This oxidative stress state further activates the NLRP3 inflammasome. This promotes the release of downstream inflammatory cytokines IL-1β and IL-18. These cytokines thereby exacerbate vascular endothelial inflammation. Additionally, PM_2.5_ exposure downregulates vascular endothelial growth factor (VEGF) expression. This impairs the ability of endothelial cells to form tubular structures. Consequently, normal vascular repair and physiological function are compromised [112].

Multiple studies have simultaneously indicated that exposure to COFs has a significant impact on the body’s oxidative stress status and lipid metabolism balance. A survey of food service workers conducted by Zhou et al. found that COF exposure can elevate serum lipid peroxides and blood lipid levels, disrupting the body’s redox balance, as evidenced by increased MDA levels and a pattern of superoxide dismutase (SOD) activity that initially rises and then declines. More importantly, excess free radicals not only form adducts with biomacromolecules (the molecular basis of genetic damage) but also oxidatively modify low-density lipoprotein (LDL). Oxidized LDL cannot be recognized by normal LDL receptors and is instead taken up by macrophages, thereby significantly increasing the risk of cardiovascular diseases such as atherosclerosis and coronary heart disease [113]. A study by Zhu et al. further confirmed that PM_2.5_ from cooking fumes induces oxidative stress by elevating ROS levels and decreasing SOD activity, which in turn inhibits the VEGF/VEGFR2/MEK1/2/ERK1/2/mTOR signaling pathway, providing molecular-level evidence that COF exposure mediates lipid metabolism disorders and cardiovascular disease risk through oxidative stress [111].

(4) Inflammation of adipose tissue

Wang et al. systematically investigated the regulation of inflammation in adipose tissue by COFs using an acute exposure mouse model [114]. Healthy female C57BL/6J mice were divided into a control group and two exposure groups (3 days and 7 days). The results showed that COF exposure significantly promoted inflammatory responses. Serological analysis indicated that pro-inflammatory cytokines (e.g., IL-6, IL-27, and IL-1β) in the exposed mice increased in a time-dependent manner (*p* < 0.05). This suggests that COF exposure can induce systemic inflammation. Flow cytometry further revealed a significant increase in the proportions of CD4^+^ and CD8^+^ T cells in adipose tissue (*p* < 0.05). The CD4^+^/CD8^+^ ratio also increased with prolonged exposure. These findings indicate that T cell subset imbalance contributes to the COF-induced inflammatory process. Molecular mechanism studies revealed that COF exposure significantly upregulated the mRNA and protein expression of NF-κB, NLRP3, Caspase 1, and IL-1β in adipose tissue (*p* < 0.05). This suggests that activation of the NF-κB/NLRP3 inflammasome pathway is a key molecular event in COF-induced adipose tissue inflammation. This study elucidated the specific mechanism by which COFs promote adipose tissue inflammation through regulation of the NLRP3/Caspase 1/IL-1β signaling pathway [114].

### 5.5. Health Risk Assessment Models for Cooking Oil Fume Exposure

(1) Toxic Equivalent Quantity Model for PAHs

The TEQ model for PAHs is a toxicity assessment approach based on the reference standard of benzo[a]pyrene (BaP), which is used to quantify the integrated carcinogenic risk of complex PAH mixtures. By introducing Toxic Equivalency Factors (TEFs), this model normalizes the toxic effects of different PAH monomers into BaP-equivalent toxicity values. The calculation formula is as follows [115,116]:

$$TEC_{i}=C_{i}*TEF_{i}$$

where *TEC* denotes the benzo[a]pyrene-equivalent concentration of the PAH congener (units: μg/kg/μg/m^3^); *C* represents the concentration of the PAH congener; *TEF* refers to the toxic equivalency factor of the PAH congener relative to benzo[a]pyrene (dimensionless).

(2) Lifetime Cancer Risk Assessment

Lifetime Cancer Risk (LCR) assessment is a quantitative risk evaluation method used to estimate the probability of an individual developing cancer over a lifetime due to prolonged exposure to carcinogenic pollutants [75,115,117]. This model integrates exposure parameters and toxicological data to calculate carcinogenic risk. The fundamental formula is as follows:

$$EC=C*\left[(ET*EF*ED)/AT\right]$$

$$LADD=\left(C*IR*ET*EF*ED\right)/\left(BW*AT\right)$$

LCR=LADD∗CSF

where *LCR* denotes the lifetime cancer risk, a dimensionless quantitative estimate of carcinogenic probability; *CDI* represents the chronic daily intake (unit: mg/kg-day); *SF* refers to the cancer slope factor (unit: (mg/kg-day)^−1^); *C* indicates the concentration of the target pollutant (unit: µg/m^3^); *ET* is the exposure time (unit: h/day); *EF* signifies the exposure frequency (unit: days/year); *Ed* defines the exposure duration (unit: years); *AT* corresponds to the averaging time, calculated as 70 years × 365 days/year × 24 h/day; *IR* represents the inhalation rate (unit: m^3^/h); *BW* indicates the body weight (unit: kg).

Risk interpretation is performed as follows: LCR < 1 × 10^−6^, no significant carcinogenic risk to humans; 1 × 10^−6^ ≤ LCR ≤ 1 × 10^−4^, potential carcinogenic risk to the population; LCR > 1 × 10^−4^, carcinogenic risk is present, with higher values indicating greater risk.

(3) Non-Carcinogenic Risk Assessment

Non-carcinogenic risk assessment assesses potential adverse health outcomes (e.g., organ damage, reproductive or developmental toxicity) arising from prolonged exposure to non-carcinogenic contaminants, including heavy metals and volatile organic compounds [5,118,119].

$$\begin{array}{l} HI=CDI_{\mathrm{nc}}/R\mathrm{fc}\\ HI_{Tot.}=\sum HI_{i} \end{array}$$

where *HQ* denotes the hazard quotient (dimensionless); *CDI* represents the chronic daily intake via inhalation (unit: mg/m^3^); *Rfc* refers to the reference concentration in air (unit: mg/m^3^), which represents the long-term safety threshold for pollutant exposure.

Risk is interpreted as follows: HQ ≤ 1, exposure level is below the threshold of adverse effects, indicating low non-carcinogenic risk; HQ > 1, exposure exceeds the safety threshold, suggesting elevated non-carcinogenic risk that requires attention.

(4) ICRP Respiratory Deposition Model

The International Commission on Radiological Protection (ICRP) Respiratory Deposition Model is a mathematical framework established by the ICRP to quantitatively estimate the deposition distribution of aerosol particles of different sizes in the human respiratory system. This model has been widely applied in health risk assessments of radioactive particles and environmental inhalable particulate matter (PM) [120,121]. The deposition fraction in each region is calculated as follows:

$$RD_{s}=PM*IR*DF$$

$$DF_{HA}=\left[1-0.5\left(1-\left(1/\left(1+0.0076d_{p}^{2.8}\right)\right)\right)\right]*\left(\left[1/\left(1+\exp(6.84+1.183\ln d_{p})\right)\right]+\left[1/\left(1+\exp(0.924-1.885\ln d_{p})\right)\right]\right)$$

$$DF_{TB}=\left(0.00352/d_{p}\right)*\left[\exp\left(-0.2341{\left(\ln_{dp}+3.40\right)}^{2}\right)+6.39\exp\left(-0.819{\left(\ln d_{p}-1.61\right)}^{2}\right)\right]$$

$$DF_{AL}=\left(0.0155/d_{p}\right)*\left[\exp\left(-0.416{\left(\ln d_{p}+2.84\right)}^{2}\right)+19.11\exp\left(-0.482{\left(\ln d_{p}-1.362\right)}^{2}\right)\right]$$

where *PM* denotes the ambient particulate matter concentration (unit: μg/m^3^); *IR* represents the inhalation rate, with a default value of 1.25 m^3^/h adopted for light activity in adults; *DF* indicates the deposition fraction characterizing the efficiency of particle deposition in specific regions of the respiratory tract, comprising: *DF_HA_* for the head airways, *DF_TB_* for the tracheobronchial region, *DF_AL_* for the alveolar region; *d_p_* refers to the particle aerodynamic diameter (unit: μm).

## 6. Advances in Key Factors and Control Strategies for Cooking Oil Fume Formation

During food preparation, the interaction between oils and ingredients initiates complex chemical reactions. The high-temperature cooking methods characteristic of Chinese cuisine led to a multifaceted mechanism governing the formation of COFs, which is influenced by various factors [122]. This section systematically reviews recent advances in the key factors affecting COFs formation and the corresponding regulation strategies, aiming to provide a theoretical basis for reducing cooking-related pollution.

### 6.1. Effects of Edible Oil Characteristics and Cooking Conditions on the Generation of Cooking Oil Fumes

The generation of COFs during culinary processes is a complex physicochemical dynamic process, whose emission characteristics and formation mechanisms are subject to the interactive effects of multiple factors. Elucidating the action principles of these influencing factors is not only the scientific basis for revealing the formation mechanisms of COFs but also a crucial prerequisite for formulating precise and efficient emission reduction strategies. Based on this, this section systematically reviews the effects of cooking process parameters and edible oil properties on COFs generation. Table 2 compares the differences among different research methods, Table 3 summarizes the cooking temperature ranges for different edible oils, Table 4 summarizes the specific effects of key influencing factors on cooking oil fume generation, and Table 5 summarizes the generation characteristics of target compounds in cooking oil fumes under different cooking conditions. This aims to provide a theoretical basis for understanding and controlling cooking oil fume pollution from the source.

#### 6.1.1. Influence of Cooking Conditions on Cooking Oil Fume

(1) Temperature and Time

Cooking temperature and duration are critical factors regulating the generation of COFs [132]. Giuffer et al. analyzed the heating processes of four edible oils, including extra virgin olive oil (EVOO), pomace olive oil (PO), palm oil (P), and soybean oil (SO). Significant differences in the VOCs produced by different oil types were observed [123]. EVOO primarily generated E-2-hexenal (28.3%), PO produced decanal (42.54%) and E-2-hexenal (16.01%) as its main components, P emitted nonanal (32.75%) and Z-2-decenal (6.82%), while SO mainly released nonanal (32.75%) and decanal (34.19%). Furthermore, the results demonstrated that heating temperature and treatment duration exerted a highly significant influence on the total aldehyde content of the oil samples. Taking EVOO as an example, the relative content of total aldehydes exhibited a continuous accumulation trend with increasing thermal treatment intensity: it increased significantly from 32.1% in the initial state to 60.9% after heating at 180 °C for 120 min, and further rose to 68.8% after heating at 220 °C for 120 min [123]. Further investigation by Zhang et al. explored the role of temperature in PM emissions [126]. When heated at 190 °C, 220 °C, and 260 °C, the PM_1.0_ mass concentration from edible oil showed a temporal profile characterized by a steep increase at the onset and then a slow decay. The peak concentration and decay time showed positive correlation, and the emission factor (EF) of PM_1.0_ increased significantly from 0.056 mg/(g·h) to 3.981 mg/(g·h) as the temperature rose. In addition, the study by Xie and Gao provided strong evidence from an engineering control perspective [129]. Their systematic investigation focused on the minimum exhaust flow rate required by residential kitchen range hoods to effectively capture cooking fumes when oil surface temperatures approached its smoke point. It was found that the exhaust flow rate required for effective fume capture rose exponentially as the pan surface temperature increased from 200 °C to 300 °C. For instance, under high-temperature stir-frying conditions (close to 300 °C), the required exhaust flow rate was more than twice the rate needed for lower-temperature cooking (below 200 °C). The primary reason is that high temperatures promote the initial ejection velocity of oil fumes, consequently increasing the total mass of pollutants released. Additionally, high temperatures alter the thermodynamic properties of the fume, thereby increasing the challenge of their effective capture. From the perspective of pollutant control efficiency, these findings indirectly confirm that cooking temperature is a core parameter determining the intensity of COF emissions. In addition, considering the potential for various thermochemical reactions in food, excessively high heating temperatures can also generate multiple endogenous risk factors in thermally processed foods themselves (such as heterocyclic amines, acrylamide, chloropropanol esters, and glycidyl esters), thereby affecting food quality.

(2) Cooking Methods

Different cooking methods vary significantly in temperature, duration, and oil contact. The temperature range for pan-frying and shallow-frying is primarily 150–180 °C. In contrast, deep-frying can range from 150 to 210 °C. For duration, pan-frying typically takes 2–5 min. Shallow-frying takes 5–10 min. Deep-frying takes 2–10 min. For oil contact, pan-frying uses only a small amount of oil to coat the pan bottom. This allows the food to remain partially in contact with the pan surface. During shallow-frying, the food is partially submerged in oil. Deep-frying, however, requires the food to be completely submerged in a large volume of oil [133,134,135,136]. The generation of PM_2.5_ in kitchen environments is significantly influenced by the cooking method employed. This is primarily attributable to fundamental variations in thermal processing temperatures, the quantity of edible oil used, and the specific modes of interaction between heat, oil, and ingredients across different techniques [130]. A comparative study by Lan et al. using the same ingredient (eggs) demonstrated that PM_2.5_ emission concentrations vary by orders of magnitude depending on the cooking method. Steaming, which uses water vapor as the heat transfer medium, resulted in the lowest PM_2.5_ concentration range (0.079–0.414 mg/m^3^, which is 5.3–27.6 times higher than the WHO air quality guidelines), while frying and stir-frying, which rely on high-temperature oil, reached 0.081–25.9 mg/m^3^ (5.4–1726.7 times the WHO air quality guidelines) and 0.528–26.4 mg/m^3^ (35.2–1760.0 times the WHO air quality guidelines), respectively [131]. Compared to steaming and boiling, stir-frying, pan-frying, and deep-frying produce higher levels of particulate matter and volatile compounds. This trend is not unique to Chinese cooking; similar phenomena can be observed in Western and other Asian culinary practices. For instance, deep-frying in Western cuisines and high-temperature stir-frying in Thai and Vietnamese cooking also generate higher PM_2.5_ and VOC emissions compared to boiling or steaming [137]. This indicates that high-temperature oil-based cooking methods have significantly higher particulate matter emission potential across different cuisines. Furthermore, the influence of cooking methods is reflected not only in particulate matter concentration but also in the formation of multiphase composite pollution. A study by Lu et al. on VOC emissions and exposure characteristics in Chinese household cooking highlighted that high-temperature frying and stir-frying are major sources of high-risk VOCs such as aldehydes and ketones [119]. These findings are consistent with results from Western cooking settings, where grilling and pan-frying have also been shown to produce similar aldehydes and heterocyclic amines [138]. Such gaseous pollutants readily adsorb onto the surface of PM_2.5_, forming organic–particulate composite systems that exacerbate health risks. Therefore, the assessment of cooking pollution should shift from a single particulate metric to a multipollutant perspective, an approach that is applicable across different dietary cultures. Low-temperature water-based cooking methods (such as steaming and boiling) not only effectively suppress PM_2.5_ generation but also avoid the formation of composite pollutants associated with high-temperature reactions. In contrast, high-temperature oil-based cooking methods—whether in Chinese, Western, or other Asian kitchens—require integrated source control and end-of-pipe purification measures to mitigate their environmental and health impacts.

#### 6.1.2. Effects of Cooking Oil Properties on Cooking Oil Fume

(1) Refining Degree

The refining grade of edible oils significantly influences the formation and emission of hazardous components such as particulate matter, PAHs, and carbonyl compounds during high-temperature cooking [139]. This influence is exerted through several mechanisms: altering fatty acid composition, removing impurities, and reducing the content of micronutrients that might otherwise mitigate harmful reactions. Studies indicate that the effect of refining on pollutant emissions exhibits notable temperature dependence. Within low to medium temperature ranges (<220 °C), oil refining effectively reduces pollutant emissions. For instance, degumming reduces particulate matter concentration by 65.67% at 190 °C [105]. Neutralization substantially decreases benzo[a]pyrene content (reduction of 64.29–98.61%) within 190–220 °C. Bleaching and deodorization also help lower the background levels of PAHs [140]. However, under high-temperature conditions (>250 °C), refined oils may promote pollutant generation due to the loss of natural antioxidants (e.g., Vitamin E) and altered oxidation pathways. Deodorization at 250 °C increases BaP concentration by 1.47 times, while the total PAHs concentration at 280 °C surges by 375.76 times compared to that at 190 °C [105]. Excessively high deodorization temperatures may trigger thermal cracking of oil, generating new PAHs [140]. Simultaneously, the ability of refined oils to suppress carbonyl compounds is significantly weakened at high temperatures. The impact of refining grade on edible oil safety is dual: it facilitates emission reduction during low- to medium-temperature cooking but may increase the risk of PAHs generation and carbonyl compound formation during high-temperature cooking. Therefore, a comprehensive safety assessment must account for the actual cooking temperatures employed.

(2) Fatty Acid Composition of Oil

The fatty acid profile of edible oils plays a critical role in determining COF formation. Zhang et al. [125] demonstrated that, relative to animal fats (e.g., lard, butter) characterized by high saturated fatty acid content, most vegetable oils, which are rich in unsaturated fatty acids, release considerably higher levels of VOCs upon heating. This phenomenon is attributed to the fact that a higher degree of unsaturation renders fatty acids more susceptible to thermal degradation and oxidative reactions. The underlying mechanism is that greater unsaturation of fatty acids promotes oxidative decomposition upon heating, thereby generating more volatile hazardous emissions [125]. Research by Li et al. further supports this finding and provides a deeper mechanistic explanation [141]. Through a systematic analysis of polar lipid component variations in frying oils possessing different fatty acid compositions throughout the frying process, the study demonstrated that unsaturated fatty acids, especially polyunsaturated fatty acids, are more susceptible to oxidative cleavage under high-temperature conditions. This process not only generates more polar compounds (e.g., carbonyl compounds such as aldehydes and ketones) but also further promotes oil deterioration, thereby increasing volatile organic compound emissions. This mechanism directly explains why oils with high unsaturation generate more cooking pollutants during heating and repeated use. These studies confirm a significant positive correlation between the degree of unsaturation in edible oils and the emission intensity of COF pollutants. Animal fats (e.g., lard) exhibit lower pollutant emission characteristics under identical heating conditions due to their high saturated fatty acid content and low degree of unsaturation.

### 6.2. Strategies for Controlling Cooking Oil Fume Generation at Source and During Processing

Given that cooking activities constitute a major source of indoor organic pollutants and pose potential threats to human health and environmental quality, the implementation of effective control strategies is of critical importance. Based on a synthesis of existing research, the mitigation pathways for COFs can be primarily categorized into two main approaches: source control and process intervention. Source control aims to fundamentally reduce the generation potential of pollutants through the optimization of their precursors, whereas process intervention focuses on suppressing the dispersion of pollutants after their formation and reducing their concentration in the indoor environment through physical means. Accordingly, this section systematically explores the influence of key factors on COF emissions and their optimization strategies from the dual perspectives of source control and process intervention. The aforementioned regulatory factors, along with their corresponding control mechanisms and optimization strategies, are summarized in Table 6.

#### 6.2.1. Source Control Strategies for Cooking Oil Fume

(1) Fuel Type

The type of fuel has a decisive influence on particulate matter emissions during cooking, with different heating systems exhibiting significant differences in emission characteristics. Existing studies indicate that traditional solid fuels (e.g., coal and biomass) produce substantial incomplete combustion products during burning, resulting in significantly higher particulate matter emission levels compared to modern clean energy systems. In contrast, gaseous fuels (including natural gas and liquefied petroleum gas), owing to their high combustion efficiency and mature pollutant control technologies, effectively reduce the emission intensity of fine particulate matter such as PM_2.5_. Compared to combustion-based methods, electric heating systems entirely avoid combustion processes, theoretically achieving zero direct particulate emissions during cooking and demonstrating superior environmental performance. Where practical kitchen conditions permit, prioritizing electric cooking equipment could significantly reduce indoor particulate matter pollution load [141,142].

(2) Impact of Cooking Temperature

A clear dose–response relationship exists between cooking temperature and pollutant emissions. As the cooking temperature increases from 190 °C to 260 °C, the EF of PM_1.0_ rises significantly from 0.044 mg/(g·h) to 3.981 mg/(g·h) [126], while the generation concentration of TVOCs presents an approximately ten-fold increase (from 1.735 to 18.519 mg/m^3^) [124]. This trend is primarily attributed to the intensification of lipid oxidative cleavage reactions under high temperatures. Research by Dong et al. further supports this conclusion from the perspective of regulating COF generation through cooking temperature [149]. By comparing the generation concentrations of PM_2.5_ during the frying process with different edible oils at temperatures ranging from 180 °C to 200 °C, the study found that PM_2.5_ emissions significantly increased with rising cooking temperatures for both unrefined rapeseed oil and peanut oil. Furthermore, repeated heating cycles were also found to markedly elevate pollutant emissions. This finding corroborates the aforementioned studies, collectively indicating that high-temperature cooking substantially promotes the formation and release of particulate matter. Based on the experimental data, controlling the cooking temperature below 200 °C could significantly reduce pollutant emissions while ensuring food safety and culinary outcomes.

(3) Ingredient Properties

The physicochemical properties of ingredients, particularly moisture and fat content, are the key factors that significantly influence the generation of COFs. Under high temperatures, the rapid vaporization of surface moisture easily causes oil splattering and particulate matter formation. Concurrently, high-fat ingredients (e.g., beef, pork, chicken) release substantial amounts of oil during heating, significantly increasing the risk of secondary smoke generation. Zhao et al. explicitly stated in their review that the physical characteristics (e.g., particulate concentration and size distribution) and chemical composition of cooking emissions are primarily regulated by the type of food, with high moisture and high fat content as the core ingredient properties leading to increased emissions [143]. Targeted pre-treatment measures (e.g., surface dehydration of ingredients) could effectively reduce COF emissions by 20–30% [144]. Therefore, appropriate surface treatment (e.g., moisture control, oil absorption) of high-moisture or high-fat ingredients before cooking could significantly lower pollutant emissions.

(4) Edible Oil Selection

The release characteristics of COFs in edible oils depend largely on their fatty acid composition, refining level, and antioxidant components [150,151,152]. Highly refined edible oils, due to the removal of free fatty acids, pigments, and unstable components during processing, exhibit superior thermal stability, thereby effectively suppressing the generation of particulate matter and VOCs during high-temperature cooking. This conclusion is highly consistent with the findings of Zhao et al., whose review clearly identified the type and quality of oil (including refining grade) as one of the core factors regulating the characteristics of cooking emissions [143]. The refining process, removing impurities (such as free fatty acids), could increase the smoke point, thereby significantly reducing smoke and particulate matter generation during heating. Mehany et al. investigated the role of hydroxytyrosol (HTyr) during deep-frying. Their findings indicate that HTyr, as a natural phenolic antioxidant, can significantly improve the thermal stability of extra virgin olive oil and other edible oils, affecting both chemical composition and sensory quality. On one hand, HTyr effectively protects monounsaturated fatty acids (e.g., oleic acid) and slows their degradation. On the other hand, it inhibits lipid rancidity and preserves positive sensory attributes, such as fruitiness, bitterness, and pungency [127,128]. Additionally, Cheng et al. investigated the inhibitory effects of β-carotene and astaxanthin on PAH formation. They found that both antioxidants significantly reduced PAH yield and their toxicity equivalents [153]. It is recommended to prioritize the use of refined cooking oils in food preparation, and to choose oils that are as rich as possible in antioxidants [105,126].

#### 6.2.2. Process Control Strategies for Cooking Oil Fume

(1) Kitchen Space Design

Kitchen volume exhibits a significant negative correlation with the concentration of particulate matter generated during cooking. Residential kitchens in China are typically designed within a volume range of 4–8 m^2^ [154]. Experimental data show that, under constant ventilation conditions, increasing the kitchen volume from 8 m^3^ to 18 m^3^ could reduce the peak and average particulate matter concentrations by 55.56%, confirming the dilution effect of spatial volume on pollutants [144]. The study by Kong et al. provides important environmental empirical support for this spatial dilution effect [155]. Monitoring in real residential kitchen environments revealed that under insufficient or inactive ventilation, confined kitchen spaces lead to extreme accumulation of PM_2.5_, with concentrations exceeding 760 μg/m^3^. This, conversely, underscores the importance of spatial volume as a key environmental design parameter for controlling pollutant concentrations. The dilution effect stems from the physical reduction in pollutant concentration per unit volume. Based on this, residential design should consider appropriately increasing kitchen volume, with a recommended minimum volume standard of above 15 m^3^, to fully leverage the spatial dilution effect and effectively reduce residents’ exposure to particulate matter during cooking [144].

(2) Ventilation Enhancement and Auxiliary Equipment Optimization

Studies indicate that enhanced ventilation could effectively reduce particulate matter concentrations generated during cooking [131]. Experiments by Zhang demonstrated that increasing the air exchange rate (AER) significantly lowers PM_1.0_ concentration and accelerates its decay. When AER increased from 1 h^−1^ to 5 h^−1^, the peak and average PM_1.0_ concentrations decreased by 53.34% and 64.01%, respectively, and the time to decay to background levels was shortened by approximately 50% [144]. Lan et al. investigated the synergistic emission reduction effects of range hoods and auxiliary facilities. After activating the range hood, the peak PM_2.5_ concentrations for steaming eggs, stir-frying shredded potatoes, and frying chicken wings decreased from 0.335 mg/m^3^ to 0.026 mg/m^3^, 28.710 mg/m^3^ to 6.712 mg/m^3^, and 29.095 mg/m^3^ to 0.743 mg/m^3^, respectively [131]. Installing baffles, air curtains, or their combination further reduced PM_2.5_ concentrations by 7.7–71.29%, with the combined use of baffles and air curtains yielding the best results. Through numerical simulations, Xin et al. further revealed that maintaining range hood operation for 2 min after turning off the stove could effectively control peak particulate matter concentrations [156]. However, the practical promotion of these ventilation enhancement technologies requires comprehensive consideration of their economic feasibility, household applicability, and user acceptance. Increasing the air exchange rate or installing auxiliary devices such as baffles and air curtains often involves high retrofit costs, space requirements, and operational noise issues. These factors may limit their application in ordinary household kitchens. Furthermore, user acceptance of delaying range hood shutdown after cooking is constrained by concerns over additional energy consumption and convenience of use. Therefore, future research should strengthen techno-economic assessments and user behavior surveys for typical household scenarios. This will help identify integrated ventilation solutions that are cost-effective, easy to install, and likely to be adopted by users over the long term.

#### 6.2.3. End-of-Pipe Control

(1) Application of Adsorption Technology in VOCs Purification

Due to its high efficiency and controllability, adsorption has become a primary method for VOCs purification. This method removes VOCs from cooking fumes by trapping them on the surface of porous adsorbent materials through physical or chemical interactions [145]. Studies have shown that adsorbent performance directly determines purification efficiency. Common adsorbents include activated carbon, metal–organic frameworks (MOFs), and mesoporous silica. Kim et al. investigated the adsorption and catalytic oxidation performance of amorphous manganese dioxide/activated carbon (Am-MnO_2_-AC) composites for formaldehyde and toluene. At room temperature, the 10% breakthrough volumes for formaldehyde and toluene were 45.9 L·g^−1^ and 152 L·g^−1^, respectively. These values represent an approximately 270–280-fold increase compared to pure activated carbon. Furthermore, Am-MnO_2_-AC exhibited high catalytic activity. It could completely oxidize formaldehyde at 100 °C and toluene at 275 °C into CO_2_ [157]. Fan et al. synthesized a chromium-containing ordered mesoporous silica material (CrSBA-15) using a co-assembly method. The study found that at a Si:Cr ratio of 30, the material had the largest micropore volume and exhibited excellent adsorption performance for toluene, benzene, cyclohexane, and ethyl acetate. At 25 °C, its static adsorption capacity for toluene was 0.92 mmol·g^−1^, and its dynamic adsorption capacity for 1000 ppm toluene was 0.72 mmol·g^−1^. Furthermore, compared to activated carbon, CrSBA-15 is easier to recover. This demonstrates its broad application prospects for VOC adsorption and removal [158]. Hu et al. synthesized methyl/phenyl-functionalized SBA-15 materials through acid-mediated modification. Their work further confirmed that functionalized mesoporous silica significantly enhances adsorption for aromatic compounds [159]. These studies provide an important technical pathway for developing highly efficient and economical VOC adsorption materials.

(2) Application of Catalytic Conversion Technology in VOC Treatment

Catalytic conversion technologies include two main approaches: catalytic combustion and photocatalytic degradation. Catalytic combustion converts VOCs into harmless substances (e.g., CO_2_ and H_2_O) at relatively low temperatures using a catalyst. The key factor is catalyst selection. The main catalyst types include precious metal-supported catalysts, transition metal oxide catalysts, and composite metal catalysts. Ying et al. prepared CeO_2_/Cu_2_O composite materials on copper mesh using a wet chemical method. This material exhibits strong photogenerated electron migration capability and can be used for VOC photocatalytic oxidation. Under light irradiation, a 15 cm^2^ CeO_2_/Cu_2_O mesh completely degraded toluene, xylene, and formaldehyde, each at an initial concentration of 50 ppm. The degradation times were 70 min, 50 min, and 6 min, respectively [146]. These results demonstrate that the CeO_2_/Cu_2_O catalyst has great practical potential for indoor VOC removal. Liu et al. prepared layered double hydroxide (LDH) as a support to load Pt nanoparticles. These were then coated onto CeO_2_ nanoparticles to construct the Pt-LDH/CeO_2_ catalyst. This catalyst exhibited excellent photothermal synergistic catalytic performance for toluene oxidation. Under visible light irradiation, its toluene conversion rate reached 75.7% [160]. Kong et al. used a hydrothermal method to incorporate Pt and Bi into Bi_2_WO_6_/CeO_2_. This successfully constructed a Pt/Bi-BWO/CeO_2_ catalyst. Under visible light irradiation, this catalyst exhibited highly efficient and stable photocatalytic degradation of typical VOCs. Its reaction rates were 20.2 times higher than those of commercial CeO_2_ and 2.3 times higher than those of BWO/CeO_2_ [161]. Catalytic conversion technology achieves high VOC removal efficiency under laboratory conditions. It also shows great potential for air purification. However, this technology faces several challenges. These include high catalyst costs, large reactor sizes, and the limited applicability of UV light sources or heating devices in residential settings. Therefore, this technology is more feasible for industrial settings. Its application in residential settings remains limited.

(3) Application of Biological Purification Technology in VOC Treatment

Biological purification technology uses microbial metabolism to efficiently degrade VOCs. Based on this principle, Yan et al. constructed a biofilter with corn cob activated carbon as the packing material. This filter removed grease and TVOCs from kitchen fumes. At a residence time of 3.24 s, the system’s maximum removal capacity was 112–235 g/(m^3^·h). At a packing material moisture content of 70%, the TVOC removal rate was 88% ± 4%. At 76% moisture content, the grease removal rate was 90% ± 3%. Microbial community analysis indicated that Thermobacillus was the dominant bacterial genus in the packing material [147]. Hu et al. used natural loofah as packing material for a biological scrubber. They used it to domesticate activated sludge and form a biofilm. This enhanced the purification capacity for cooking fumes. After the loofah biofilm matured, the biomass reached 104.56 mg/g. The removal rates for grease, non-methane total hydrocarbons, PM_2.5_, and PM_10_ were 91.53%, 67.53%, 75.25%, and 82.23%, respectively. The system’s maximum removal capacity for cooking fumes was 20.7 g/(m^3^·h). Microbial diversity analysis revealed that Proteobacteria was the dominant phylum. The major genera included Sphingomonas, Mycobacterium, and Lactobacillus [162]. According to the aforementioned study, biological purification technology can effectively remove grease and TVOCs and demonstrates good purification potential. However, its applicability in residential settings is limited due to technical constraints. These constraints include large equipment size and the need for media maintenance. Residential settings typically have limited space and require ease of operation.

(4) Electrostatic Precipitation Technology in VOC Treatment

Researchers have investigated the purification efficiency of electrostatic precipitation technology for VOC treatment. This includes both plasma-coupled DC bias and conventional electrostatic precipitation for oily aerosols and gaseous pollutants. Yang et al. used a method that combines transient pulsed plasma with a DC bias to treat oil-based aerosols. Plasma discharge was generated in a 4-inch-diameter cylindrical reactor. The system used a pulse generator with an output voltage of 30 kV and a pulse width of 5–10 nanoseconds. A DC bias of up to 10 kV was also applied simultaneously. The system achieved a particulate removal efficiency of 99.9% [148]. Gysel et al. investigated the impact of electrostatic precipitation (ESP) technology on particulate matter and gaseous toxic pollutant emissions from cooking fumes. The results showed that this technology achieved a mass removal efficiency of 86–90% for PM_2.5_. It also reduced formaldehyde and acetaldehyde concentrations by 73% and 74%, respectively. Furthermore, ESP significantly reduced the emission levels of aromatic VOCs and 1,3-butadiene [163]. These results indicate that electrostatic precipitation and plasma-based purification technologies offer distinct advantages for removing particulate matter and VOCs from cooking sources. However, for domestic applications of this technology, factors such as equipment miniaturization and long-term operational safety must be carefully considered.

## 7. Current Challenges and Future Perspectives

Current research on cooking oil fumes and related pollutants exhibits the following major limitations. In terms of data and methodology, existing studies predominantly rely on laboratory simulations or commercial kitchen environments, lacking long-term systematic monitoring in household cooking scenarios, especially those involving high-temperature Chinese cooking practices. Meanwhile, the analysis of high-risk components such as ultrafine particles and sticky particulate matter remains insufficient. In terms of health risk assessment, current research largely focuses on short-term exposure effects, while the carcinogenic mechanisms of long-term low-dose exposure, population-specific risks, and associated molecular toxicity pathways remain poorly understood. From the perspective of food processing and kitchen environments, research on the synergistic effects of multiple pollutants is still scarce. Regarding control technologies, existing purification techniques exhibit low removal efficiency for nano-sized particles and polar volatile organic compounds, and their applicability in household settings is poor. Furthermore, due to the current lack of international guideline values or statutory regulatory standards regarding cooking fumes, there is a lack of clear quantitative basis for assessing the health risks of pollutants in cooking fumes and for managing them environmentally, which in turn hinders the establishment of a precise prevention and control system. This situation underscores the urgent need to expedite the development of relevant standards.

Future research on cooking fumes should prioritize the following areas to address current research gaps. First, efforts should focus on standardizing sampling and testing methods. Standardized procedures should be established for collecting and analyzing fume components across different cooking scenarios. This will enhance the comparability and reproducibility of research results. Second, long-term monitoring systems should be established for real-world home kitchen settings. For example, real-time monitoring of home kitchens should be conducted in different regions for at least 12 months. This monitoring should focus on key pollutants such as PM_2.5_, VOCs, and PAHs. The goal is to obtain representative exposure data. Finally, long-term population-based cohort studies should be conducted. These studies will clarify the dose–response relationship between cooking fume exposure and respiratory and cardiovascular diseases. This will provide a scientific basis for developing precise health risk assessments and protective strategies.

The core scientific challenges lie in accurately characterizing the toxicity contributions of key components, balancing the cost-effectiveness of control technologies, and modeling emission characteristics specific to the diverse range of Chinese cooking styles. Future efforts must strengthen interdisciplinary integration across environmental science, public health, food science, and materials engineering to translate fundamental research findings into precise prevention and control strategies. Such an approach will not only enhance environmental health risk management but also support broader carbon neutrality goals through targeted emission reductions.

## 8. Conclusions

In recent years, growing concerns regarding air quality and chronic health risks have brought increased attention to the health impacts of COFs. This paper systematically elucidates the physicochemical properties, formation pathways, health risks, influencing factors, and control strategies associated with COFs, and provides insights into the core scientific challenges in this field. The findings reveal that COFs are chemically complex and contain multiple hazardous components, with their generation and composition influenced by various factors (including cooking oil, ingredients, cooking temperature, cooking methods, etc.). Therefore, accurate quantification of their health risks necessitates assessment based on emission characteristics under specific cooking practices. COFs exhibit distinct characteristics—highly dispersed emission sources, low single-event emission intensity, yet extremely high emission frequency. These features render conventional end-of-pipe control measures ineffective, posing a fundamental bottleneck for management. Regarding emission reduction, balancing operational feasibility with mitigation efficiency is a prerequisite for household applications. Given the significant regional variations in Chinese cooking styles, differentiated modeling analysis tailored to diverse cooking practices represents an effective approach to enhancing health risk management. Notably, reducing COFs emissions is not only beneficial for public health but also contributes to carbon neutrality, as lower energy consumption during cooking and reduced loading on ventilation systems lead to decreased greenhouse gas emissions. Furthermore, interdisciplinary integration of environmental science, public health, and materials engineering is essential to translate fundamental insights into targeted prevention and control strategies. Such an integrated approach, by promoting source reduction and energy-efficient cooking practices, supports precise emission control while aligning with carbon neutrality pathways. The findings of this study provide a theoretical basis for improving COFs pollution management systems and offer valuable references for developing integrated strategies that jointly address air quality improvement, health risk mitigation, and carbon neutrality goals.

## Figures and Tables

**Table 1:** Hazardous components in cooking oil fume and associated health risks.

Substance Category	Representative Compound	Inhalation Toxicity	Health Endpoints	Reference Source
Aromatic Hydrocarbons	Benzene	Lymphocytopenia	Carcinogenicity, Leukemia	EPA
Aldehydes	Formaldehyde	Decreased pulmonary function	Non-carcinogenic risk	EPA
Ketones	2-Butanone	Developmental toxicity (skeletal variations)	Non-carcinogenic risk	EPA
PAHs	Benzo[a]pyrene	Squamous cell tumors in throat, respiratory tract, and esophagus	Carcinogenicity	EPA
Particulate Matter	PM_2.5_	Lung deposition inducing pulmonary dysfunction	Non-carcinogenic risk	[94] (2019); [95] (2018)

Note: The United States Environmental Protection Agency (EPA).

**Table 2:** Influencing Factors of COFs Generation.

Influencing Factors	Effect on Cooking Oil Fume Production	Key Mechanism	References
Temperature	PM: Emission concentration and emission factor (EF) exhibit a significant increase with rising temperature. VOCs: Total concentration and hazardous components show a marked rise with increasing temperature.	High temperature intensifies lipid oxidation, cracking, and volatilization.	[73]
Time	Within a given duration, both the peak concentration and the cumulative emission increase with extended heating time	Prolonged reaction time is associated with an increase in cumulative pollutant levels.	[123]
Cooking method	Steaming/Boiling: Characterized by low emissions of PM and VOCs with a simple pollutant profile. Stir-frying/Pan-frying/Deep-frying: Characterized by high emissions of PM and VOCs, prone to forming complex, multiphase pollution.	Differences in heat transfer medium and maximum cooking temperature	[119]
Refining degree	At low to medium temperatures, oil refining is beneficial for controlling COFs emissions. At high temperatures, refined oil may pose an increased risk of generating PAHs and carbonyl compounds (aldehydes/ketones).	Refining enhances oil purity but removes natural antioxidants, thereby altering the reaction pathways for the generation of hazardous components.	[105]
Composition of edible oil	A higher degree of unsaturation in edible oils leads to a significant increase in pollutant emissions.	Edible oils rich in unsaturated fatty acids exhibit a greater tendency toward oxidation and cracking reactions.	[124]
Repeated use	Grade-3 edible oils exhibit higher TVOC emissions compared to Grade-1 oils. Oil reuse significantly increases the emission of pollutants.	Lower-grade edible oils contain a higher level of impurities and an elevated initial oxidation state; repeated use leads to the accumulation of polar compounds and oxidative polymerization products, thereby exacerbating pollutant formation.	[124]

**Table 3:** Cooking Temperature Ranges for Common Cooking Oils.

Type of Edible Oil	Cooking Temperature (°C)	Cooking Methods	Cooking Time (min)	References
Soybean oil	130–280	Stir-frying, Pan-frying, Deep-frying	2–10	[105,125]
Peanut oil	130–260	Stir-frying, Pan-frying	2–10	[125,126]
Rapeseed oil	190–270	Stir-frying	2–5	[126]
Sunflower oil	190–260	Stir-frying	2–5	[125,126]
Olive oil	170–210	Tossed, Stir-frying	2–5	[127,128]
Palm oil	180–220	Deep-frying	2–10	[126]
Lard	130–270	Deep-frying	2–10	[125]

**Table 4:** Comparison of Experimental Conditions Across Different Research Methods.

Cooking Method	Types of Cooking Oils	Cooking Oil Usage	Ingredients	Temperature (°C)	Ventilation Rate	Target	References
-	Soybean oil	80 g	-	190–280	-	Particulate matter, PAHs, carbonyl compounds	[105]
Steam, boil, stew, stir-fry, deep-fry	Soybean oil	-	-	-	15–22 m^3^/min	TOVCs	[119]
-	Olive oil, palm oil, soybean oil	1 L	-	180–220	-	VOCs	[123]
-	Soybean oil, sunflower oil, peanut oil, rapeseed oil, blended oil, lard	100 g	-	190–260	-	PM_1.0_, PM_2.5_, PM_10_	[126]
-	Olive oil	160 g		200–300	200–800 m^3^/h	Particulate matter	[129]
Stir-fry, pan-fry, deep-fry	Sunflower oil, rapeseed oil, palm oil	10 mL–600 mL	Potato, pork tenderloin (160 g)	-	-	Aldehydes	[130]
Steam, stir-fry, pan-fry	-	-	Egg, potato, chicken wing	-	-	PM_2.5_	[131]
-	Rapeseed oil, soybean oil, peanut oil, corn oil, lard	-	-	130–270 °C	-	VOCs	[125]

**Table 5:** Formation Characteristics of Target Substances in Cooking Oil Fumes and Intervention Effects of Antioxidants Under Different Cooking Conditions.

Type of Edible Oil	Frying Method	Temperature Range (°C)	Heating Duration (min)	Determination of the Target Substance	Natural Antioxidants/ Effects of Carotenoids	Key Findings	References
Soybean oil	deep-frying	190–280	30	PAHs, PM, aldehydes and ketones	-	Elevated temperatures significantly increase pollutant concentrations, and the effect of purification degree is target-specific.	[105]
Olive oil, palm oil, soybean oil	deep-frying	180–220	30–120	VOCs, aldehydes and ketones	alpha-tocopherol	Heating significantly alters the composition of VOCs; the higher the temperature, the more pronounced the increase in aldehydes.	[123]
Soybean oil, sunflower oil, peanut oil, canola oil, blended oil, lard	deep-frying, stir-frying	190–260	30	PM	**-**	Particulate matter concentrations increase significantly as temperatures rise	[126]
olive oil	-	200–300	12	PM	-	Ventilation can effectively reduce particulate matter	[129]
Canola oil	deep-frying	160–280	30	aldehydes and ketones	Rapeseed polyphenols, tocopherols, and beta-carotene	As temperature increases, aldehyde and ketone levels rise significantly, while rapeseed polyphenols, tocopherols, and β-carotene markedly reduce them	[130]
Canola oil, soybean oil, peanut oil, corn oil, lard	-	130–270	-	VOCs	-	The total concentration of VOCs increases significantly as temperature rises, and oils with high levels of unsaturated fatty acids emit higher levels of VOCs.	[125]

**Table 6:** Control strategies for COFs.

Control Level	Strategy Category	Specific Measures	Key Mechanism	References
Source Control	Fuel substitution	Substitute coal or biomass with electricity or natural gas.	Eliminate particulate matter and pollutants from the incomplete combustion of solid fuels	[142]
	Cooking parameter control	Control oil temperature and reduce high-heating duration.	Reduce the reaction kinetics rate, thereby decreasing the generation intensity of thermal oxidation and cracking products.	[126]
	Ingredient properties	Drain ingredients after washing prior to cooking.	Reduce mass transfer effects and spattering during water vaporization.	[143]
	Edible oil selection	Select edible oils with high refining grade and low unsaturation, and avoid repeated use.	Enhance thermal stability, reduce impurities and oxidizable components, and lower the reaction activity of the oil.	[105]
Process Control	Kitchen space	Appropriately increase the kitchen volume.	Leverage the spatial dilution effect to lower pollutant concentration.	[144]
	Ventilation measures	Use high-performance rangehoods; ensure adequate kitchen airflow; delay turning off the hood after cooking.	Enhance pollutant dispersion and promptly remove COF components from the human breathing zone.	[131]
End-of-Pipe Treatment	Adsorption Technology	Adopt adsorbent materials such as activated carbon, zeolites, MOFs, and mesoporous silica.	Capture VOCs on the material surface via physisorption (e.g., van der Waals forces).	[145]
	Catalytic Conversion Technology	Employ catalytic combustion (using catalysts such as noble metals or metal oxides) and photocatalytic degradation.	Oxidize VOCs to CO_2_/H_2_O at lower temperatures via catalytic action, or degrade them photocatalytically.	[146]
	Biological Purification Technology	Utilize specific microbial consortia for synergistic degradation.	Microorganisms utilize VOCs as a carbon and energy source, converting them into harmless substances through metabolism.	[147]
	Electrostatic Precipitation	Capturing cooking fumes using electrostatic forces	In a high-voltage electrostatic field, the components of cooking fumes are ionized and attracted to the collection plates.	[148]

## Data Availability

No new data were created or analyzed in this study. Data sharing is not applicable to this article.

## Abbreviations

The following abbreviations are used in this manuscript:

COFs	cooking oil fumes
VOCs	volatile organic compounds
PM	particulate matter
PAHs	polycyclic aromatic hydrocarbons
O_3_	ozone
SOA	secondary organic aerosols
Dp	aerodynamic diameter
PM_0.1_	ultrafine particles
SVOCs	semi-volatile organic compounds
TG	triglycerides
DG	diglycerides
MG	monoglycerides
FFA	free fatty acids
BTEX	benzene, toluene, ethylbenzene, and xylenes
